# Young men are at higher risk of failure after ACL hamstring reconstructions: a retrospective multivariate analysis

**DOI:** 10.1186/s12891-022-05547-8

**Published:** 2022-06-21

**Authors:** Martine C. Keuning, Bart J. Robben, Reinoud W. Brouwer, Martin Stevens, Sjoerd K. Bulstra, Rutger G. Zuurmond

**Affiliations:** 1 Department of Orthopaedic Surgery, University of Groningen, University Medical Center Groningen, Postbus 30.001, 9700 RB Groningen, Netherlands; 2 Department of Orthopaedic Surgery, Isala, Postbus 10400, 8000 GK Zwolle, Netherlands; 3 Department of Orthopaedic Surgery, Martini Hospital, Postbus 30.033, 9728 NT Groningen, Netherlands

**Keywords:** ACL, Reconstruction, Failure, Graft, Hamstring

## Abstract

**Background:**

Results of ACL reconstruction are influenced by both patient and surgical variables. Until now a significant amount of studies have focused on the influence of surgical technique on primary outcome, often leaving patient variables untouched. This study investigates the combined influence of patient and surgical variables through multivariate analysis.

**Methods:**

Single-center retrospective cohort study. All patients who underwent primary ACL hamstring reconstruction within a 5-year period were included. Patient characteristics (gender, age, height, weight, BMI at time of surgery) and surgical variables (surgical technique, concomitant knee injury, graft diameter, type of femoral and tibial fixation) were collected. Patients were asked about Tegner Activity Scale (TAS), complications and revision surgery. Multivariate logistic regression was used to study risk factors. First graft failure and potential risk factors (patient and surgical) were univariately assessed. Risk factors with a *p*-value ≤ 0.05 were included in the multivariate model.

**Results:**

Six hundred forty-seven primary ACL hamstring reconstructions were included. There were 41 graft failures (failure rate 6.3%). Patient gender, age, height and preoperative TAS had a significant influence on the risk of failure in the univariate analysis. The multivariate analyses showed that age and sex remained significant independent risk factors. Patients with a failed ACL reconstruction were younger (24.3 vs 29.4 years, OR 0.937), with women at a lower risk for failure of their ACL reconstruction (90.2% males vs 9.8% females, female OR 0.123). ACL graft diameter and other surgical variables aren’t confounders for graft failure.

**Conclusion:**

This study shows that patient variables seem to have a larger influence on the failure rate of ACL hamstring reconstructive surgery than surgical variables. Identification of the right patient variables can help us make more informed decisions for our patients and create patient-specific treatment protocols. Young men’s higher risk of failure suggests that these patients may benefit from a different reconstruction technique, such as use of a patellar tendon or combined ligament augmentation.

**Level of evidence:**

Retrospective cohort III.

**Supplementary Information:**

The online version contains supplementary material available at 10.1186/s12891-022-05547-8.

## Background

Anterior cruciate ligament (ACL) surgery has evolved tremendously over the past 50 years [1, 2]. Despite these developments, the failure rate for ACL reconstruction remains relatively high [3–6]. The exact reason for the high rates is still an issue of debate. As stated below various causes are presented, mostly related to surgical technique and to a lesser extent patient characteristics [3–19].

The risk of ACL failure with hamstring autografts is reported to be 3–12% [3–6]. The majority of studies have focused on the influence of surgical technique. Some studies show greater risk of failure in the early years of anatomical ACL reconstruction [7]. The methods used for graft fixation likewise influence the risk of failure [8]. Clinical studies identify an inconsistent correlation between graft size and failure rate [9–12]. Also, concomitant injury may lead to higher instability after ACL rupture, but the influence on failure remains unclear [13].

A minority of studies have identified patient-specific predictors of failure. Failure has been associated with younger age [9–11, 14]. Other studies have investigated gender as a predictor of failure, with inconsistent results [6, 10, 15–18]. The influence of patients’ activity level on failure also remains a point of debate in literature, with studies showing that a higher activity level leads to a higher risk [19] and others showing no influence [9]. A major drawback of most of these studies is that they predominantly analyzed the influence of the potential variables univariately. Hence the purpose of this study is to analyze the combined influence of surgical and patient variables in a multivariate fashion. Our hypothesis is that patient variables have a higher influence on the failure of primary ACL hamstring reconstruction than surgical variables.

## Methods

### Population

All patients who underwent primary ACL hamstring reconstruction within a 5-year period at a single-center teaching hospital were included. Patients had a minimum follow-up of two years. Patients with ACL reconstruction other than hamstring, multiligament reconstructions and open growth plate at the time of reconstruction were excluded. Patients aged 18 and older at the time of follow-up were contacted.

### Data collection

After approval of the local Medical Ethics Committee (METC nr: 16.06105), all ACL reconstructions between 1 January 2010 and 31 December 2014 were included. Failure was defined as repeat ACL reconstruction, ACL graft failure objectified by MRI, or arthroscopic surgery. Baseline patient characteristics (gender, age, height, weight, BMI at time of surgery) and surgical variables (surgical technique, concomitant knee injury, graft diameter, type of femoral and tibial fixation) were collected from hospital records.

Patients were contacted by one of the researchers (MK) by phone, between January 1, 2017 and July 1, 2017. After obtaining consent they were asked about preoperative activity level using the Tegner Activity Scale (TAS) [20]. Patients were also asked about postoperative complications and treatments at other hospitals. The date of ACL re-rupture was determined using the questionnaire and hospital records.

### Surgical procedure

All ACL reconstructions were performed according to national guidelines, and a uniform postoperative rehabilitation protocol was prescribed for all participants [21].

Patients underwent ACL reconstruction with a semitendinosus and gracilis tendon. Due to an institutional change in treatment protocol two surgical techniques were performed. First we used a transtibial reconstruction technique (TT), for non-anatomical ACL reconstruction. The graft is fixated using the transfix on the femoral side and an interference screw on the tibial side (Arthrex Inc., Naples, FL, USA). The other technique was anteromedial portal (AMP) [22], for anatomical ACL reconstruction. The graft is fixated using an endobutton on the femoral side and an interference screw on the tibial side (Smith & Nephew, Andover, MA, USA).

### Rehabilitation

All patients received a standardized protocol for rehabilitation with clinical physiotherapy starting on day 1 postoperatively. Standard follow-up was performed 2 weeks, 6 weeks and 3 months postoperatively. After this follow-up only those patients with persisting complaints or complications visited the outpatient clinic.

### Statistical analysis

Statistical analyses were performed using IBM SPSS Statistics 24 (IBM Armonk, NY, USA). Descriptive statistics were used to describe demographic characteristics and failure rate. The Pearson chi-squared test and a Mann–Whitney U-test were conducted to determine the influence of patient and surgical characteristics on early and late failure. Logistic regression analysis was used to determine risk factors for graft failure. First graft failure and each potential risk factor (both patient and surgical) were univariately assessed. Risk factors with a *p*-value ≤ 0.05 were considered eligible for inclusion in the multivariate logistic regression analysis model (stepwise Backwards Likelihood Ratio model). As due to the limited number of ACL failures we were restricted to include a maximum of four variables in the multivariate logistic regression analysis, we opted for the four variables with the highest significance. Using a multivariate logistic regression analysis we were able to correct for missing data. We used the largest possible dataset for all variables. Additionally, we performed a sensitivity analysis between the entire ACL reconstruction group and those patients available for questionnaires. A *p*-value ≤ 0.05 was considered statistically significant.

## Results

### Population

**Fig. 1:**
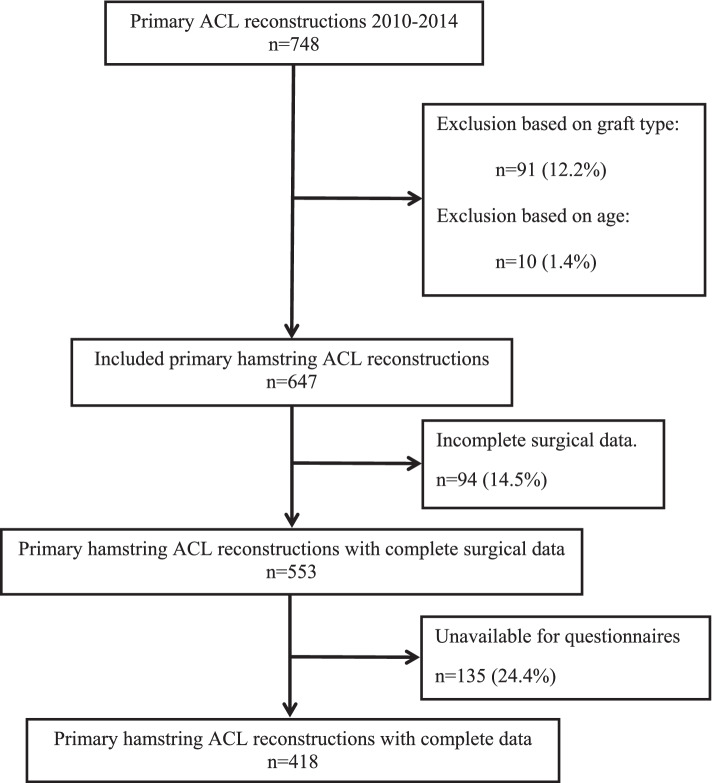
Flow chart of the numbers of patients that were excluded and included for the primary hamstring ACL reconstructions with complete data

A total of 748 ACL reconstructions were performed between 1 January 2010 and 31 December 2014. After exclusion of 101 ACL reconstructions, 647 primary ACL reconstructions (638 patients) were available for this study. Of these reconstructions 553 (85.5%) had full surgical data available, with an mean follow-up of 5.5 years, and 418 (75.6%) patients were available by phone to answer the research questionnaires (Fig. 1). All the available data from 647 primary ACL reconstructions were included in the data analysis. Table 1 displays the demographics of the patient population.

**Table 1:** Demographics of the primary ACL reconstruction at time of surgery

*N *= 647	Mean/N	(SD or percentage)
Gender		
- Male	438	(67.7%)
- Female	209	(32.3%)
Age	28.8 years	(10.6)
Height	1.79 m	(0.09)
Weight	79.7 kg	(14.4)
BMI	24.9	(4.0)
Follow-up	5.5 years	(1.5)
TAS (428) (median, range)	7	(0–10)

*BMI* Body mass index, *TAS* Tegner Activity Scale preoperatively

The sensitivity analysis between the entire ACL reconstruction group and those patients available for questionnaires only showed a significant difference between the tibial fixations.

### Graft failure

There were 41 failed ACL reconstructions (failure rate 6.3%). Table 2 displays the distribution of patient and surgical variables between failed and intact ACL reconstructions.

**Table 2:** Distribution of variables between failed and intact ACL reconstructions

Failure	No	Yes	Univariate		Multivariate	
*N* = 647	606 (93.7%)	41 (6.3%)	OR	95% CI	OR	95% CI
Gender						
Male	401 (66.2%)	37 (90.2%)	1.00			
Female	205 (33.8%)	4 (9.8%)	0.211*	0.074–0.601	0.123*	0.024–0.632
Age (years)	29.4	24.3	0.945*	0.909–0.982	0.937*	0.886–0.990
Height (cm) *N* = 591	178	182	1.049*	10.01–1.089	0.990	0.937–1.046
Weight (kg) *N* = 590	79.6	81.7	1.010	0.988–1.032		
BMI *N* = 590	25.0	24.6	0.977	0.898–1.062		
Pre-op TAS *N* = 415 (median, range)	7 (0–10)	7 (2–10)	1.429*	1.105–1.849	1.122	0.852–1.479
Concomitant injury *N* = 647						
None	255 (42.1%)	16 (39.0%)	1.00			
Cartilage	53 (8.7%)	1 (2.4%)	0.301	0.039–2.317		
Meniscus	236 (38.9%)	20 (48.8%)	1.351	0.684–2.668		
Collateral ligament	6 (1.0%)	0 (0.0%)	15.938	0.952–266.702		
Combined ^a^	55 (9.1%)	3 (7.3%)	0.869	0.245–3.086		
Graft diameter (mm) *N* = 567	8.1	8.2	1.054	0.590–1.881		
Surgical technique *N* = 577						
AMP	326 (60.4%)	25 (67.6%)	1.00			
TT	214 (39.6%)	12 (32.4%)	0.731	0.360–1.487		
Femoral fixation *N* = 638						
Endobutton	452 (75.7%)	34 (82.9%)	1.00			
Transfix	144 (24.1%)	7 (17.1%)	0.646	0.280–1.489		
Tibial fixation *N* = 629						
Screw	246 (41.6%)	21 (55.3%)	1.00			
BioScrew	345 (58.4%)	17 (44.7%)	0.577	0.298–1.117		

*CI* Confidence interval, *OR* odds ratio, *BMI* Body mass index, *TAS* Tegner Activity Scale preoperatively, *TT* Transtibial, *AMP* Anteromedial portal

^*^P values < 0.05

^a^combined meniscus and cartilage injury

Six of the 41 failed ACL reconstructions were threated in other clinics. From these 6 we couldn’t accurately determine the time of failure, due to this we allocated them as missing. From the remaining failed ACL reconstructions 18 (43%) occurred within the first 12 months after surgery, 4 (10%) between 12 and 24 months and 13 (32%) after two years.

To gain insight into the influence of the variables on the risk of failure, first an univariate analysis was conducted. Patient gender, age, height and preoperative TAS had a significant influence on the risk of failure (Table 2), with a higher number of men with a failed ACL reconstruction (90.2% males vs 9.8% females, female OR 0.123). Patients with a failed ACL reconstruction were younger (24.3 vs 29.4 years, OR 0.937), taller (1.82 vs 1.78 m, OR 0.990), and had a higher TAS (7.6 vs 6.6, OR 1.122). The surgical variables (graft diameter, surgical technique, concomitant injury, femoral fixation and tibial fixation) had no significant influence on graft failure.

The four significant variables were subsequently included in the multivariate model. Age and gender remain the only significant independent variables for graft failure (Additional file 1) – age (*p* < 0.01, OR 0.937) and gender (*p* < 0.01, OR 0.123) (Table 2), with being young posing a higher risk of graft failure and women having an eightfold lower risk of graft failure.

## Discussion

This study reports an incidence of 6.3% graft failure for single-bundle ACL hamstring reconstructions. Age and gender are the only significant independent variables for graft failure, with being young posing a slightly higher risk of graft failure and women having an eightfold lower risk of graft failure. Our incidence of ACL graft failure (6.3%) is in line with current literature. By comparison, the average range described for hamstring autograft ACL surgery is 4–14% [15, 16, 23].

In this study the 0.123 OR indicates that women have an eightfold lower risk of failure than men. There is wide discrepancy in literature when it comes to gender. Wernicke et al. also showed a higher risk of failure in male patients [18], but several other studies evidence that women are at higher risk of failure [15, 16]. It could be hypothesized that women generally receive an ACL graft larger than their native ACL, which protects them from ACL graft rupture, but this needs further evaluation.

The risk of ACL graft failure at a younger age seems to be very limited in our study, with a 0.94 OR per year. Many other studies on ACL graft failure identify younger age as a predictor for graft failure [10, 11, 14, 15, 18, 24]. This might be due to incomplete neuromuscular development.

The surgical variables in this study did not have any influence on the risk of failure. Many studies have investigated the role of surgical variables on failure rate [7–9, 12, 15, 18, 25, 26], some pointing to an increased risk of failure with AMP surgical technique compared to TT ACL reconstruction [7, 27]. Recent studies with the New Zealand ACL registry using a multivariate analysis revealed no difference in surgical technique. A Norwegian registry study shows an increased revision rate for endobutton/biosure hydroxyapatite screw fixation [8]. In the same study transfix with metal interference screw fixation had the lowest revision rate in ACL hamstring reconstruction. Although our study displays a similar trend, there was no significant difference in fixation method or surgical technique with respect to risk of failure.

Based on our results, pre-injury activity level is not a risk factor for failure after ACL surgery. This outcome is in line with the results of Yabroudi et al., evidencing higher risk of failure with participation in sports at a competitive level in a univariate analysis but no difference in a multivariate analysis [28]. In other studies activity level was found to be a risk factor, yet they used univariate analyses and no correction was done for the influence of other variables as we did in our study [19].

Graft diameter was not of significant influence for failure. Our study complements multiple others showing no correlation between graft diameter and graft failure [9, 12, 15, 18].

### Limitations of the study

Several limitations of our study should be mentioned. First of all, this is a retrospective analysis, and although we weren’t able to contact a quarter of the patients we did use their available data in the multivariate analysis. Patients were asked about instability and revision surgery, but this study is lacking a clinical score to objectify such instability − plus if there are no complaints or instability there is no need for revision surgery. Unfortunately we weren’t able asses time of return to sport and patients activity level at the last follow-up. Early return to sport or more aggressive rehabilitation may be a cause of early failure.

### Strengths of the study

Strength of the current study is that we performed a multivariate analysis that included both patient and surgical variables. Several recently published studies used multivariate analysis on ACL reconstructive surgery [24, 28]. Rahardia et al. [24] analyzed the New Zealand ACL registry, which also yielded a difference between the univariate and multivariate analyses, and with the multivariate analysis evidencing an increased risk of revision for young men.

Drawback of multivariate analysis is that it needs at least 10 cases per variable. Most randomized trials lack the number of patients and data needed to draw conclusions based on multivariate analyses. Registry studies provide more consistent data and a larger number of patients. This will hopefully allow us to demonstrate more accurate correlations between patient characteristics, surgical variables and outcome. Currently there are only a few national registries. The implementation of more national registries could lead to more insights, and registries are upcoming in different countries.

There are many risk factors for graft failure and factors as tibial slope, notch width, ongoing anterolateral rotational laxity are not included in this article. There is also evidence that patellar tendon reconstruction or reconstruction combined with lateral extra-articular tenodesis have a lower risk for graft failure than isolated ACL hamstring reconstruction [2, 29]. The fact that young men are at higher risk of failure with ACL hamstring reconstruction suggests that these patients may benefit from a different reconstruction technique.

We hope our article adds to better understanding the risk factors in ACL reconstruction and identifying those patients at risk of graft failure. Identification of the right patient variables can help us make more informed decisions for our patients and create patient-specific treatment protocols.

## Conclusions

This study shows that patient variables seem to have a larger influence on the failure rate of ACL hamstring reconstructive surgery than surgical variables. Identification of the right patient variables can help us make more informed decisions for our patients and create patient-specific treatment protocols. The fact that young men are at higher risk of failure suggests that these patients may benefit from a different reconstruction technique such as use of a patellar tendon or combined ligament augmentation.

## Supplementary Information

**Additional file 1:** Multivariate analysis of the four significant univariate variables.

## Data Availability

The datasets generated and/or analysed during the current study are not publicly available due institutional privacy guidline but are available from the corresponding author on reasonable request.

## Abbreviations

ACL: Anterior cruciate ligament
METC: Medical Ethics Committee
BMI: Body mass index
TAS: Tegner Activity Scale
TT: Transtibial
AMP: Anteromedial portal
CI: Confidence interval
OR: Odds ratio
