# Supplementary material for: Young men are at higher risk of failure after ACL hamstring reconstructions: a retrospective multivariate analysis
Source: BMC Musculoskelet Disord. 2022 Jun 21;23:598. doi: 10.1186/s12891-022-05547-8 (PMC9210756; doi:10.1186/s12891-022-05547-8)
Supplement: Supplementary file 1 — Additional file 1: Multivariate analysis of the four significant univariate variables. [file 12891_2022_5547_MOESM1_ESM.docx]

Appendix 1: Multivariate analysis of the four significant univariate variables

|  |  | B | S.E. | Wald | df | Sig. | Exp(B) | 95% CI for EXP(B) | |
| --- | --- | --- | --- | --- | --- | --- | --- | --- | --- |
|  |  |  |  |  |  |  |  | Lower | Upper |
| Step 1^a^ | Gender | -2.097 | 0.836 | 60.295 | 1 | 0.012 | 0.123 | 0.024 | 0.632 |
|  | Height | -1.009 | 20.825 | 0.127 | 1 | 0.721 | 0.365 | 0.001 | 920.562 |
|  | Tegner | 0.115 | 0.141 | 0.673 | 1 | 0.412 | 10.122 | 0.852 | 10.479 |
|  | Age | -0.066 | 0.028 | 50.338 | 1 | 0.021 | 0.937 | 0.886 | 0.990 |
|  | Constant | 0.575 | 5.460 | 0.011 | 1 | 0.16 | 1.777 |  |  |
| a. Variable(s) entered on step 1: gender, height, Tegner, ege  CI = confidence interval  B = nonstandardized coefficients Beta  SE = nonstandardized coefficients standard error  Wald = Wald test  Df = default  Sig = significant  Exp = expecting | | | | | | | | | |
